# Oxidative Oligomerization of DBL Catechol, a Potential Cytotoxic Compound for Melanocytes, Reveals the Occurrence of Novel Ionic Diels-Alder Type Additions

**DOI:** 10.3390/ijms21186774

**Published:** 2020-09-15

**Authors:** Manickam Sugumaran, Kubra Umit, Jason Evans, Rachel Muriph, Shosuke Ito, Kazumasa Wakamatsu

**Affiliations:** 1 Department of Biology, University of Massachusetts, Boston, MA 02125, USA; Kubra.Umit001@umb.edu; 2 Department of Chemistry, University of Massachusetts, Boston, MA 02125, USA; Jason.evans@umb.edu (J.E.); Rachel.Muriph001@umb.edu (R.M.); 3 Department of Chemistry, Fujita Health University School of Medical Sciences, Toyoake, Aichi 451-0052, Japan; sito@fujita-hu.ac.jp (S.I.); kwaka@fujita-hu.ac.jp (K.W.)

**Keywords:** rhododendrol toxicity, raspberry ketone, 3,4-dihydroxybenzalacetone, DBL catechol, ionic Diels-Alder addition, melanotoxicity, leukoderma, skin lightening compounds

## Abstract

The exposure of human skin to 4-(4-hydroxyphenyl)-2-butanone (raspberry ketone, RK) is known to cause chemical/occupational leukoderma. RK is a carbonyl derivative of 4-(4-hydroxyphenyl)-2-butanol (rhododendrol), a skin whitening agent that was found to cause leukoderma in skin of many consumers. These two phenolic compounds are oxidized by tyrosinase and the resultant products seem to cause cytotoxicity to melanocytes by producing reactive oxygen species and depleting cellular thiols through *o*-quinone oxidation products. Therefore, it is important to understand the biochemical mechanism of the oxidative transformation of these compounds. Earlier studies indicate that RK is initially oxidized to RK quinone by tyrosinase and subsequently converted to a side chain desaturated catechol called 3,4-dihydroxybenzalacetone (DBL catechol). In the present study, we report the oxidation chemistry of DBL catechol. Using UV–visible spectroscopic studies and liquid chromatography mass spectrometry, we have examined the reaction of DBL catechol with tyrosinase and sodium periodate. Our results indicate that DBL quinone formed in the reaction is extremely reactive and undergoes facile dimerization and trimerization reactions to produce multiple isomeric products by novel ionic Diels-Alder type condensation reactions. The production of a wide variety of complex quinonoid products from such reactions would be potentially more toxic to cells by causing not only oxidative stress, but also melanotoxicity through exhibiting reactions with cellular macromolecules and thiols.

## 1. Introduction

Raspberry ketone (4-(4-hydroxyphenyl)-2-butanone, RK), is a phenolic compound widely used as a cosmetic, perfume and food flavoring agent [1]. Some of the workers engaged in the manufacturing process of this compound in Japan developed occupational leukoderma [2]. In this context, it is important to draw particular attention to rhododendrol, 4-(4-hydroxyphenyl)-2-butanol, the derivative of RK with its carbonyl group reduced to a secondary alcohol. Rhododendrol was also used widely in the cosmetics industry as a skin whitening product. Repeated application of rhododendrol as a skin color lightening agent resulted in the development of leukoderma on the face, neck, and hands [3]. These two compounds may be interconvertible in the cell by oxidation-reduction reactions. Therefore, their toxicity may be caused by the same kind of reactions. 

**Figure 1:**
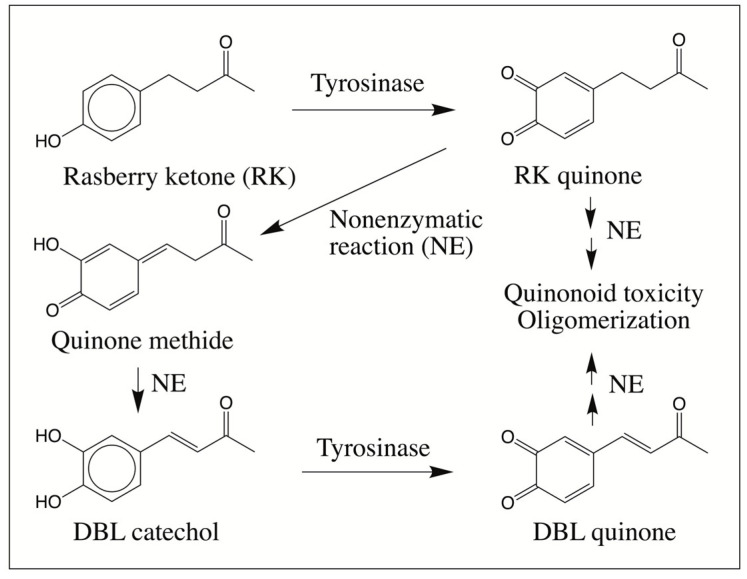
Proposed fate of raspberry ketone (RK). Tyrosinase performs facile oxidation of RK to RK quinone. This quinone causes depletion of thiols and exhibits cellular toxicity. In addition, it also undergoes nonenzymatic isomerization to DBL catechol through the intermediate formation of isomeric quinone methide. DBL catechol is further oxidized to its quinone either by tyrosinase or nonenzymatically by RK quinone. The DBL quinone thus formed is more reactive than the saturated RK quinone and hence it too exhibits cellular toxicity. It also seems to undergo polymerization to more toxic compounds (NE = nonenzymatic reaction).

The melanotoxicity exhibited by rhododendrol has been shown to occur *via* tyrosinase dependent mechanism [4,5]. Accordingly, tyrosinase was able to cause the facile conversion of rhododendrol to its *o*-quinonoid product [6,7]. The quinonoid product(s) caused reactive oxygen species production and cytotoxicity by exhibiting reaction with cellular thiols [8,9,10]. Similar studies on RK yielded valuable information on the reaction course of this compound (Figure 1). 

Tyrosinase-catalyzed oxidation of RK produced its corresponding quinone which exhibited rapid isomerization via its quinone methide to (E)-4-(3,4-dihydroxyphenyl)-3-buten-2-one, commonly known as 3,4-dihydroxybenzalacetone (DBL catechol) [11]. Such nonenzymatic introduction of the double bond in the side chain by the intermediary formation of quinone and quinone methide is a well-documented reaction for a few catecholamine derivatives [12,13,14,15]. Preliminary studies indicated that the resultant DBL catechol is very reactive and the quinonoid products formed from this compound could rapidly react with cellular thiol compounds [11]. In addition, possible oligomerization of the quinonoid product was inferred but was not examined due to extreme reactivity of the intermediates. In recent years, one of our laboratories has successfully used mass spectral studies to investigate the intricate details of oxidative transformations of a number of dehydrodopa and dehydrodopamine derivatives [12,13,14,15,16,17,18,19,20]. Our explorations yielded valuable information on the course of oxidative transformation of a number of catecholamine derivatives. Therefore, we decided to investigate the reaction course used by DBL catechol also using this technique and report in this paper the novel oxidative transformations exhibited by DBL quinone.

## 2. Results and Discussion

**Figure 2:**
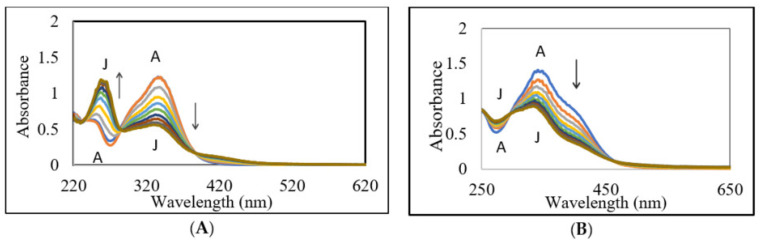
Ultraviolet and visible spectral changes associated with the tyrosinase catalyzed oxidation of DBL catechol. A reaction mixture (1 mL) containing 25 µg of mushroom tyrosinase and DBL catechol in 50 mM sodium phosphate buffer, pH 6.0 (**A**) or pH 8.0 (**B**) was incubated at room temperature and the absorbance changes occurring at UV and visible regions were followed using a spectrophotometer. For pH 6.0, the reaction was monitored at one min intervals (**A**) Scan A, start of the reaction; Scan J, 9 min reaction). For pH 8.0, the reaction was monitored at two min intervals (**B**) Scan A, start of the reaction; Scan J, 18 min reaction).

Figure 2A shows the UV-visible spectral changes accompanying the oxidation of DBL catechol by mushroom tyrosinase. As soon as tyrosinase is added, rapid changes occur in the absorbance spectra. The UV peak due to DBL catechol at around 320 nm progressively reduced and absorbance in the visible region at about 420 nm increased steadily. The spectral changes accompanying this transformation exhibited two isosbestic points at about 285 nm and 385 nm, indicating the direct transformation of DBL catechol to the compound exhibiting absorbance at about 420 nm. This compound, which has yellow color, must be the corresponding quinone as tyrosinase is well known to exhibit wide substrate specificity and oxidize many *o*-diphenolic compounds to their corresponding *o*-quinones [15]. When the reaction was performed at slightly alkaline conditions (at pH 8), the color of the reaction mixture became lighter as the reaction progressed (Figure 2B). At the end, a compound exhibiting broad absorbance at around 330 nm was formed. One could also witness a slight shift in the spectral scans between the substrate and the end product, suggesting that part of the conjugation in the substrate is lost in the product. These results indicated that the tyrosinase-generated DBL quinone is undergoing further transformation.

**Figure 3:**
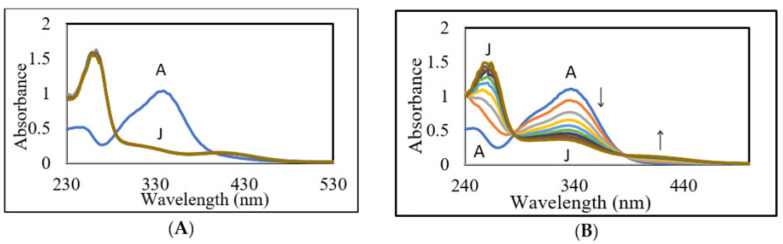
Oxidation of DBL catechol by sodium periodate in (**A**) sodium phosphate buffer, pH 7.0 and (**B**) 0.2 M acetic acid. A reaction mixture (1 mL) containing DBL catechol and equimolar amounts of sodium periodate in (**A**) 50 mM sodium phosphate, pH 7.0 or (**B**) 0.2 M acetic acid was incubated at room temperature and the absorbance changes occurring at UV and the visible region were followed using a spectrophotometer at 6 s intervals. The reaction occurred very fast and seems to have reached completion in about 6 s. Scan A, start of the reaction; Scan J, 54 s reaction.

In order to visualize the fate of DBL quinone, we generated this quinone by quantitatively oxidizing DBL catechol with sodium periodate and monitoring the UV and visible absorbance spectral changes. Initial studies conducted at pH 6 or 7 revealed that the oxidation is extremely fast and resulted in the formation of a final product which exhibited absorbance maximum at around 265 nm with a broad absorbance at visible region between 380 and 440 nm (Figure 3A).

**Figure 4:**
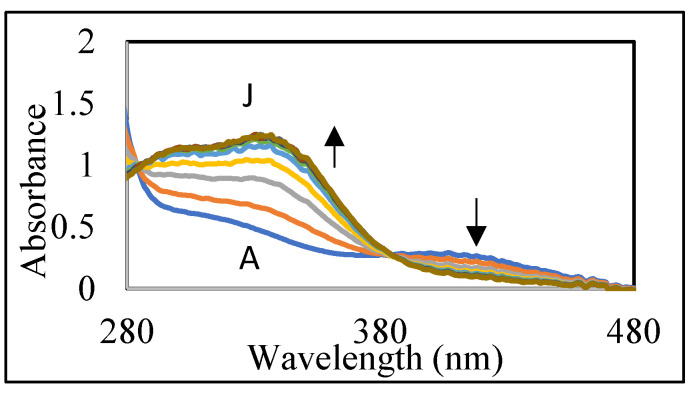
Fate of DBL quinone produced in acetic acid. The reaction conditions are the same as outlined for Figure 3B except for the monitoring the absorbance changes at 10 min intervals instead of 6 s intervals. Scan A, start of the reaction; Scan J, 90 min reaction.

Since the reaction occurred too fast, monitoring the spectral changes became very difficult. Hence, we decided to slow down the reaction by conducting it at acidic pH values. To slow the reaction and monitor the quinone formation, we conducted the studies in 0.2 M acetic acid. Figure 3B shows the rapid generation of DBL quinone from DBL catechol by the oxidation of sodium periodate in acetic acid. The oxidation was completed in less than a min and the quinone formed could be visualized by its absorbance at visible region. However, the quinone turned out to be very unstable and exhibited rapid conversion to product(s) that exhibit ultraviolet absorbance at 280–480 nm range as shown in Figure 4. 

**Figure 5:**
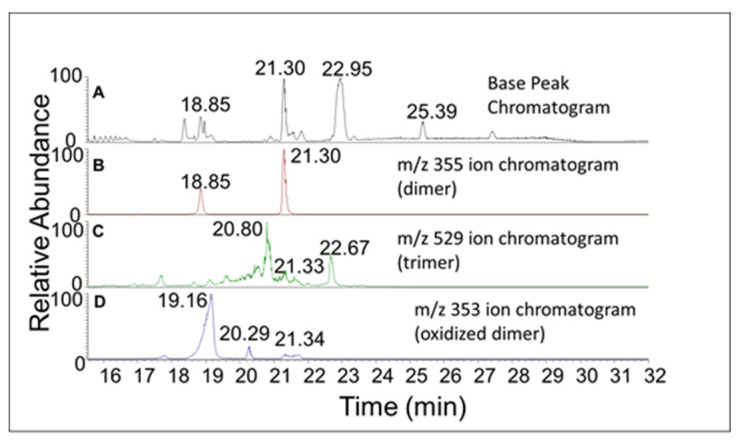
The base Peak and ion chromatograms of DBL catechol reaction products. The RP-HPLC/ESI-MS/MS base peak chromatogram produced during the incubation of DBL catechol with tyrosinase. (**A**) shows the base peak chromatogram. The ion chromatograms of dimeric, trimeric, and oxidized forms of the dimeric species are presented in (**B**–**D**) respectively.

This reaction is accomplished by the bleaching of the quinone and appearance of UV absorbing peaks at 295 and 330 nm (Figure 4). These kinds of broad absorbing peaks indicate the presence of conjugated groups to the aromatic ring. Thus, the quinone is exhibiting rapid transformation to colorless compounds possessing side chain desaturation. Extensive studies carried out on a number of catechols possessing side chain desaturation such as 1,2-dehydro-*N*-acetyldopamine [16,17,18], *N*-acetyl-1,2-dehydro dopa [19], and *N*-acetyl-1,2-dehydro dopa methyl ester [20] indicated their facile conversion to dimeric and other polymeric compounds. Hence, we envisaged that a similar reaction may also be occurring with DBL catechol upon oxidation. In order to prove this hypothesis, we performed mass spectral studies. Figure 5A shows the base peak chromatogram obtained from RP-HPLC/ESI-MS/MS analysis of the oxidation products of DBL catechol after 10 min incubation with mushroom tyrosinase. The additional panels in Figure 5B–D show the ion chromatograms of the protonated precursor product ions corresponding to the dimeric, trimeric, and oxidized form of the dimeric species, respectively. A cursory glance at these ion chromatograms reveals the generation of multiple products in each case.

**Figure 6:**
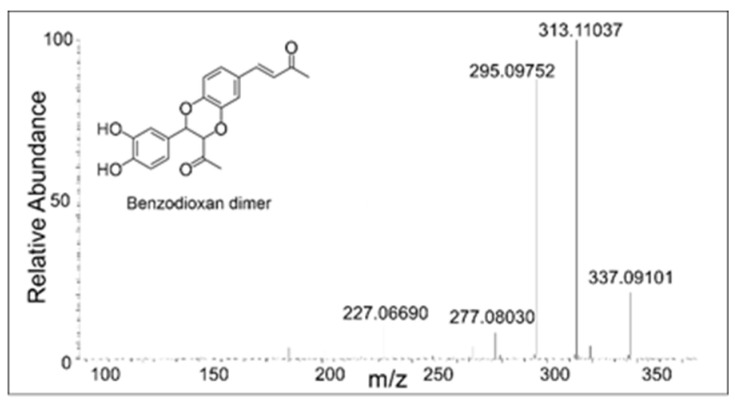
The collision induced decomposition (CID) mass spectrum of the *m*/*z* 355 mass unit ion eluting at 18 min. Its possible structure is indicated.

**Figure 7:**
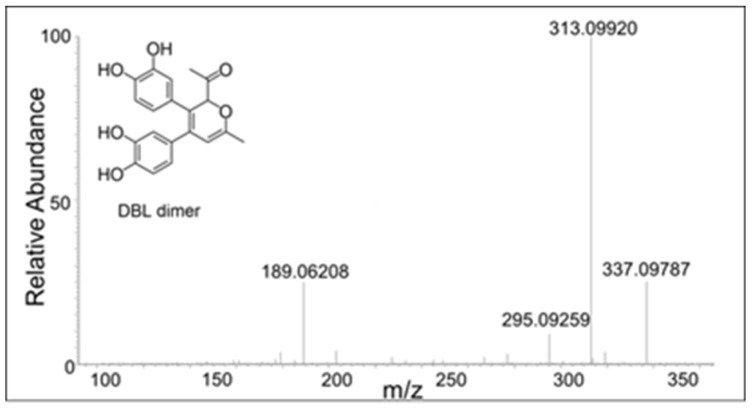
The CID mass spectrum of the *m*/*z* 355 mass unit ion eluting at 21 min. Its possible structure is indicated.

Two products—one eluting at 18 min and another eluting at 21 min—showed a mass of *m*/*z* 355.1171 (Figure 5B), which is within 3 ppm of the theoretical mass for the addition product of DBL quinone to DBL catechol (C_20_H_18_O_6_). The CID spectrum of the dimer eluting at 18 min (Figure 6) differed significantly from that eluting at 21 min (Figure 7). The dimer eluting at 18 min exhibited major product ions at *m*/*z* value of 337 (loss of water), 313 (loss of acetyl group), and 295 (loss of water and acetyl group). The loss of both water and acetyl groups is possible only for the benzodioxan type dimer shown in the inset. Note this *m*/*z* 295 ion peak is not the prominent peak in the CID spectrum of the 21 min peak. This observation coupled with the product ion observed at 189 (loss of a catecholic group and CH_2_=CO-CH_3_ group) suggests that the 21 min peak is due to a different dimer whose proposed structure is shown in the inset of Figure 7. 

**Figure 8:**
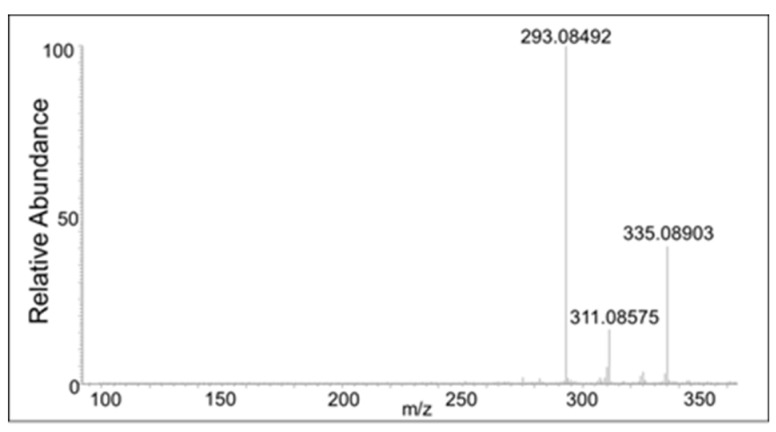
The CID mass spectrum of the *m*/*z* 353 mass unit ion eluting at 17 min.

**Figure 9:**
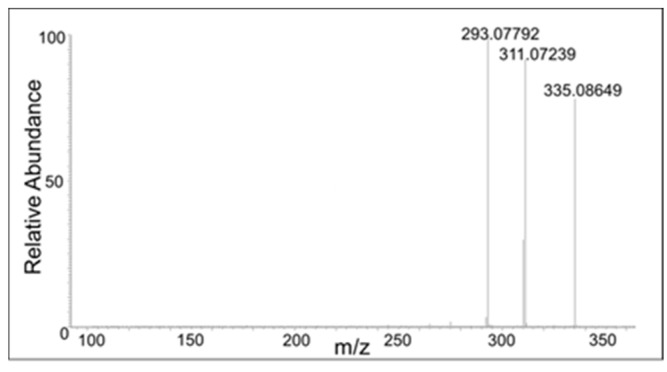
The CID mass spectrum of the *m*/*z* 353 mass unit ion eluting at 18 min.

**Figure 10:**
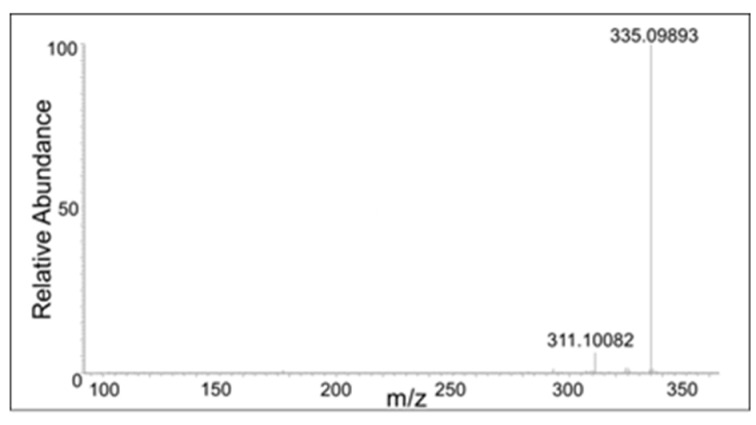
The CID mass spectrum of the *m*/*z* 353 mass unit ion eluting at 20 min.

**Figure 11:**
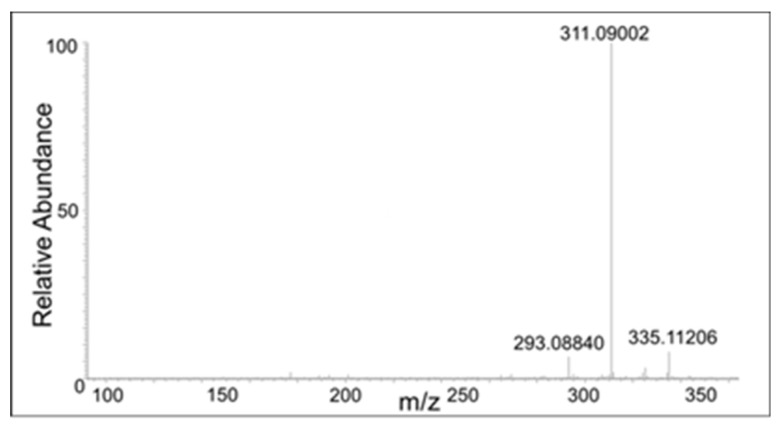
The CID mass spectrum of the *m*/*z* 353 mass unit ion eluting at 21 min.

There were also compounds formed from the dimer with the loss of two protons. These compounds eluted at 17 min, 18 min, 20 min, and 21 min with a molecular mass of 353.1021, which is within 1.5 ppm of the theoretical mass for C_20_H_16_O_6_ (353.1013 amu). The CID spectrum of these compounds were significantly different, indicating multiple isomers are being formed in the reaction mixture (Figure 8, Figure 9, Figure 10 and Figure 11).

The peak eluting at 20 min showed only a water loss as the major product ion (*m*/*z* 335 ion in Figure 10). The peak eluting at 21 min showed the major peak with the loss of COCH_2_ group (*m*/*z* 311 ion). This compound has to be the oxidized form of the DBL quinone dimer. On the other hand, the peak eluting at 18 min showed major decomposition ions at 335 (water loss), 311 (COCH_2_ loss), and a minor ion at *m*/*z* 293 (water and COCH_2_ loss). Note that the last decomposition ion is not possible for the DBL quinone dimer and is possible only for the oxidized form of the benzodioxan dimer. From these results it was inferred that two different kinds of dimers are formed in the reaction—a benzodioxan dimer and a DBL quinone dimer. 

These oxidized dimers will exhibit visible absorbance, much like any other simple unconjugated quinones. This is consistent with the absorbance maximum of 420 nm, which is due to the quinonoid compound accumulating in the DBL catechol–tyrosinase reaction mixture. The DBL quinone dimer could aromatize and further oxidize to the quinone methide. Whereas the benzodioxan dimer will undergo oxidation to quinone which will then isomerize to side chain desaturated compound. Thus, the initial dimers with *m*/*z* 355 are getting converted to the oxidized form of the dimers with *m*/*z* 353. 

**Figure 12:**
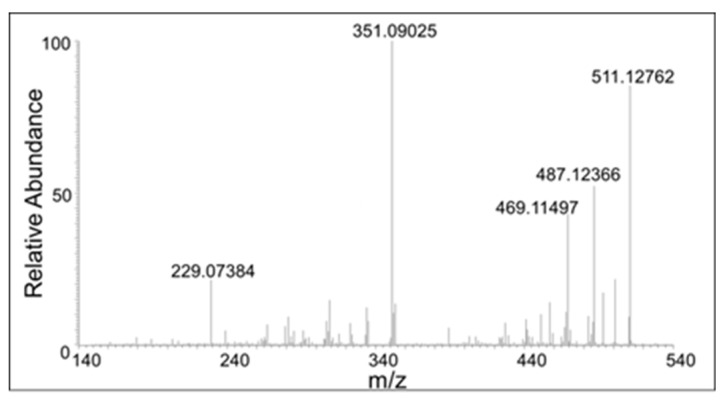
The CID mass spectrum of the *m*/*z* 529 mass unit ion eluting at 20 min.

**Figure 13:**
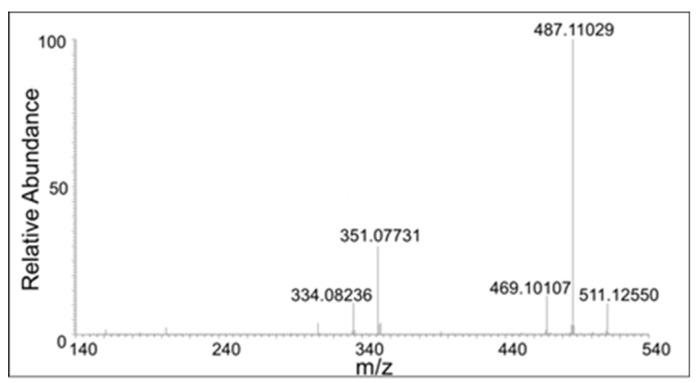
The CID mass spectrum of the *m*/*z* 529 mass unit ion eluting at 22 min.

In addition to the dimeric products, trimeric compounds could also be observed in the mass spectrum of the reaction mixture. Again, two parent ions at *m*/*z* 529.1486 are present, one eluting at 20 min and the other at 22 min (Figure 5 panel C). Their mass is within 3 ppm of the mass of the theoretical protonated trimeric compound (C_30_H_26_O_9_). Their CID spectra are shown in Figure 12 and Figure 13. The CID of one isomer gave major ion at 351, which corresponds to the fully oxidized form of the dimer. The other isomer gave considerably less amount of this product ion. It was not possible to distinguish the structure of the trimers based on the fragmentation pattern. Nevertheless, it was definitely clear that different trimeric products are also formed in the reaction mixture. Thus, the results presented in this paper confirm the DBL catechol is extremely susceptible to oxidative polymerization as proposed in an earlier work from one of our groups [11]. 

**Figure 14:**
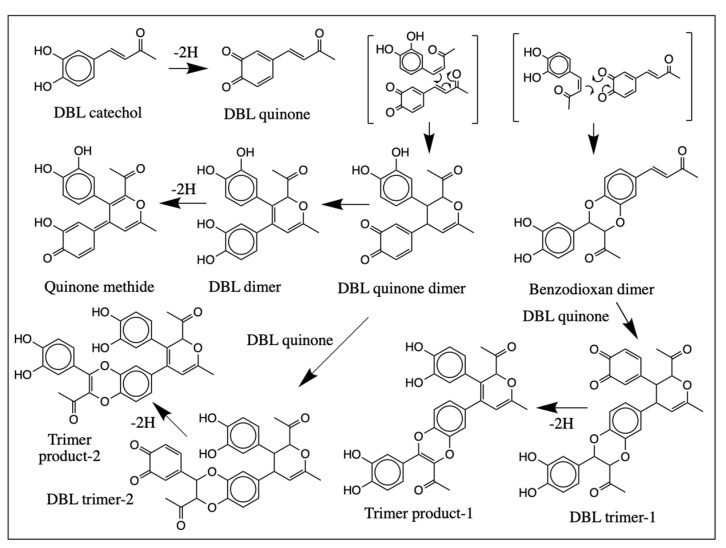
Proposed mechanism for the oxidative transformation of DBL catechol. Oxidation of DBL catechol produces the corresponding quinone which is very reactive and reacts instantaneously with the parent compound forming dimers, probably via ionic Diels-Alder addition reactions. The further oxidation of these dimers and coupling to another molecule of DBL quinone produces trimeric products. These additions are all nonenzymatic and hence, non-stereoselective multiple isomeric products are generated in each case.

The formation of dimers and trimers can be explained by the reactivities of the quinonoid products formed in the reaction (Figure 14). Oxidation of DBL catechol produces its corresponding quinone, which is highly hydrophobic and can easily exhibit cycloaddition reaction with the parent catechol. The ionic Diels-Alder addition of the DBL quinone to the parent catechol will produce two types of adducts as shown in Figure 14. The reaction of quinonoid carbonyl groups with the desaturated side chain will produce the benzodioxan dimer, while the dienone side chain addition with the desaturated side chain produces the pyran type adduct simple designated as DBL quinone dimer. Both these compounds can undergo facile oxidation and further reaction to form trimeric compounds by similar Diels-Alder reaction. Although the biological occurrence of Diels-Alder reaction is very rare, it has been reported to proceed in a few circumstances [20,21,22,23]. For example, one of our groups has recently shown that the quinone of *N*-acetyl dopa methyl ester is undergoing rapid cycloaddition, probably via ionic Diels-Alder reaction, generating a similar benzodioxan dimer [20]. The current studies also support the prevalence of such ionic Diels-Alder additions in the quinonoid chemistry of side chain desaturated catechols. Obviously, these cyclization reactions are all nonenzymatic and hence will be non-stereoselective, leading to the production of multiple isomeric products. The production of such multiple products during the nonenzymatic cyclization of enzymatically generated quinonoid species has been well documented from this laboratory for a number of dehydrodopa and dehydrodopamine derivatives [16,17,18,19,20].

The potent melanotoxicity of RK and its reduced product, rhododentrol, is now well established [1,2,3,4,5,6,7,8,24]. While some of the reactions such as the depletion of thiols and addition to cellular nucleophiles are also common to other cytotoxic quinones, the unique melanotoxicity of RK and rhododentrol could be ascribed to their ability to exhibit multiple redox reactions that produces not only their corresponding quinonoid derivatives, but also a number of side chain desaturated quinonoid species. In addition, a plethora of dimeric and trimeric compounds are produced, all with the ability to cause reactive oxygen species production, depletion of cellular thiols, and reaction with cellular macromolecules including proteins and DNA [11,24]. Compounds exhibiting such multiple redox reactions will therefore be more toxic than simple quinonoid compounds. It is rather difficult to pinpoint one or any other products of RK or rhododentrol as a causative agent for inducing leukoderma and other melanotoxic effects. With these results in mind, we caution the use of these compounds and other related catechols which have the potency to exhibit multiple redox reactions for the treatment of any melanin related disorders. 

## 3. Materials and Methods 

*Materials:* DBL catechol was procured from Fujifilm-Wako Pure Chemicals (Osaka, Japan). Mushroom tyrosinase (specific activity 5771 units/mg of protein) was purchased from Sigma Chemical Co., St. Louis, MO. HPLC grade methanol and ammonium formate (99%) were obtained from Acros, Morris Plains NJ. Milli Q synthesis A10 Water purification system purchased from Millipore, Milford, MA was used to prepare HPLC grade water. Mobile phase solvents (formic acid, acetonitrile) for mass spectrometry were purchased from Fisher Chemical (Fair Lawn, NJ, USA) and were Optima LC/MS Grade. All other chemicals were of analytical grade purchased from Fisher and/or VWR. 

*Enzyme assays:* A reaction mixture (1 mL) containing DBL catechol (usually 0.2 mM), about 5–10 µg of mushroom tyrosinase in 50 mM sodium phosphate buffer at specified pH was incubated at room temperature and the spectral changes associated with the oxidation was followed using a diode array spectrophotometer. Some reactions were conducted in acidic conditions. Chemical oxidation of DBL catechol with sodium periodate was conducted in a mole to mole ratio at specified pH values. Exact conditions are given under each figure legend. 

*Sample preparation for mass spectral studies:* A reaction mixture containing 100 nmol of DBL catechol and 5 µg of tyrosinase was incubated in 1 mL of water at room temperature for two min and an aliquot of the reaction (100 mL) was quenched with (900 mL) 1% trifluoroacetic acid. This diluted mixture was subjected to mass spectrometric analysis. The diluted reaction was directly injected into the mass spectrometer. 

*RP-nLC/ESI-MS conditions:* An Orbitrap Fusion Lumos mass spectrometer (Thermo Fisher, San Jose, CA, USA) coupled online to an EASY-nLC 1200 (Thermo Fisher, San Jose, CA, USA) was used to detect and characterize the products. The nLC system was operated at a flow rate of 300 nL/min using a linear gradient of 0–70% B in 15 min. Mobile phase A was 96.1:3.9 0.1% formic acid in water/0.1% formic acid in acetonitrile. Mobile phase B was 80.0:20.0 0.1% formic acid in water/0.1% formic acid in acetonitrile. The sample was first desalted on a Thermo Fisher Scientific Acclaim PepMap 100 C_18_ HPLC column (3 μm particle size, 75 μm × 2 cm, 100 Å) prior to separation on a Thermo Fisher Scientific PepMap RSLC C18 EASY-Spray Column (3 μm particle size, 75 μm × 15 cm, 100 Å).

The Orbitrap Fusion Lumos mass spectrometer was operated in the small molecule mode. The global settings were as follows: ion source type NSI, positive voltage of 1900 V, and an Ion Transfer Tube Temp of 275 °C. Ions for the MS scans were detected in the Orbitrap with a resolution of 30,000. The mass range was normal, and the scan range was set to 100–1000 *m*/*z*. The RF lens was set to 30% and the AGC target and maximum injection time were 4.0 × 10^5^ and 50 ms, respectively. The data-dependent MS^2^ CID scans were run in conjunction with a targeted mass filter in which the targeted masses corresponded to the following protonated species: DBL (179.0708 *m*/*z*), DBL-quinone (177.0551 *m*/*z*), DBL-quinone dimer (355.1182 *m*/*z*), DBL-quinone trimer (529.1499 *m*/*z*), DBL-water adduct (197.0813 *m*/*z*), and DBL-dimer with a loss of 2H (353.1026 *m*/*z*). An intensity threshold of 2.0 × 10^3^ was set on each mass with a mass tolerance of ± 10 ppm. Ions for the ddMS^2^ CID were isolated in the ion trap with a rapid scan rate and with an isolation window of 2 *m*/*z*. Ions were fragmented via CID with a fixed collision energy of 40%. The Q parameter for the CID activation was set to 0.25. The AGC target and maximum injection time were set to 1.0 × 10^4^ and 500 ms. The cycle time for the data-dependent acquisition was set to 3 s.

## Abbreviations

CID	Collision induced decomposition
DBL	3,4-dihydroxybenzalacetone
LC/MS	High pressure liquid chromatography/mass spectrometry
RK	Raspberry ketone
